# Risk factors for Q fever incidence in Korea: a comparative analysis using frequentist and Bayesian methods

**DOI:** 10.4178/epih.e2025046

**Published:** 2025-08-20

**Authors:** Ji-Hyun Son, Sung-Dae Park

**Affiliations:** 1 FMD and Large Animal Health Control Division, Ministry of Agriculture, Food and Rural Affairs, Sejong, Korea; 2 Planning and Finance Division, Ministry of Agriculture, Food and Rural Affairs, Sejong, Korea; 3 Graduate School of Public Administration, Korea University, Sejong, Korea

**Keywords:** Q fever, *Coxiella burnetii*, Bayesian analysis, One Health, Epidemiology, Zoonotic disease

## Abstract

**OBJECTIVES:**

This study investigated the principal determinants of human Q fever incidence and explored regional variation between metropolitan cities and provinces in Korea.

**METHODS:**

Panel data on human Q fever incidence, livestock populations, and facility metrics were collected across 17 metropolitan cities and provinces from 2017 to 2024. Analytical approaches included frequentist models (ordinary least squares [OLS], random effects [RE], fixed effects [FE]) and Bayesian models.

**RESULTS:**

Frequentist panel analysis indicated that slaughterhouse count was positively associated with Q fever incidence in both pooled OLS (β=1.20, p<0.001) and RE models (β=1.03, p<0.001), but not in the FE model (β=0.14, p=0.65). After correcting for serial correlation using Driscoll-Kraay standard errors, livestock population (β=0.55, p<0.01), livestock market count (β=-2.01, p<0.05), and livestock Q fever cases (β=-0.11, p<0.01) were significantly associated with human incidence. A Bayesian FE model confirmed a significant relationship between slaughterhouses and human Q fever incidence (posterior mean: 0.87; 95% credible interval [CrI], 0.21 to 1.42), providing more stable inference with limited samples and allowing probabilistic uncertainty estimation. A Bayesian hierarchical model revealed a stronger association in metropolitan cities (posterior mean, 1.46; 95% CrI, 0.34 to 2.57) than in provinces (1.22), while livestock population remained significant in provinces (0.94; 95% CrI, 0.15 to 1.74).

**CONCLUSIONS:**

In Korea, slaughterhouse density was the main determinant of Q fever in metropolitan cities and livestock density was the primary risk factor in provinces. These findings underscore the need for region-specific preventive strategies and reinforce the value of a One Health approach.

## GRAPHICAL ABSTRACT

[Fig f1-epih-47-e2025046]

## Key Message

This study identified that the primary risk factors for Q fever in Korea vary by region. Slaughterhouse density was the main determinant of human Q fever incidence in metropolitan cities, whereas livestock density was the primary risk factor in provinces. These findings underscore the need for region-specific preventive strategies based on a One Health approach, which integrally considers human, animal, and environmental health.

## INTRODUCTION

Zoonotic diseases account for approximately 60% of all infectious diseases, and 75% of newly emerging human infectious diseases originate from animals [1-3]. In Korea, various zoonotic diseases continue to be reported, with Q fever emerging as one of the most rapidly increasing infectious diseases in recent years [4].

Epidemiologically, the prevalence and incidence of Q fever vary substantially by region, time period, and infection source. A systematic review published in 2024 reported 81 outbreaks across 27 countries between 1990 and 2022, ranging from 2 to as many as 4,107 cases. Large-scale epidemics were predominantly linked to ruminant livestock—particularly sheep, goats, and cattle—with sheep identified as the most frequent source (28 of 81 outbreaks), followed by goats (12) and cattle (7) [5]. In Korea, Q fever became a nationally notifiable infectious disease in 2006, with an annual average of about 10 reported cases. However, according to the Infectious Disease Portal of the Korea Disease Control and Prevention Agency (KDCA), the national incidence rate per 100,000 population peaked at 0.31 in 2018, before declining and stabilizing at 0.09-0.13 from 2020 to 2024.

Q fever is caused by infection with the bacterium *Coxiella burnetii*, primarily transmitted through the milk, urine, and birthing materials of infected cattle, sheep, and goats [6]. Human infection occurs mainly via inhalation of aerosolized bacteria derived from the excreta of infected animals. Other transmission routes, such as tick bites, ingestion of unpasteurized milk or dairy products, and human-to-human transmission, are rare. Therefore, most Q fever outbreaks and sporadic cases are associated with contact with livestock or their environments rather than direct tick exposure [7]. Previous studies have identified individuals in livestock-related occupations, including livestock workers, farmers, abattoir employees, veterinarians, and laboratory staff, as high-risk groups [8]. For example, a study conducted by the New South Wales Department of Agriculture in Australia found that 10.7% of veterinarians and livestock inspectors tested positive for Q fever antibodies [9,10]. In Korea, the KDCA conducted a 2020 seroprevalence survey of Q fever among goat farm workers, confirming a high prevalence among goat and sheep farm workers.

Approximately 5% of acute Q fever cases progress to chronic Q fever, and failure to promptly treat acute cases increases the likelihood of chronic disease. Chronic Q fever carries a mortality rate of approximately 65% and often results in severe complications such as endocarditis [11,12]. Because about 50% of Q fever infections are asymptomatic, the risk of missed diagnoses and delayed treatment is high, emphasizing the need for proactive disease management [13,14].

Since Q fever affects both humans and animals, effective prevention and control require a multidisciplinary approach integrating human, animal, and environmental health perspectives [15]. In Korea, previous studies have primarily examined Q fever seroprevalence among abattoir and farm workers or assessed the correlation between livestock population density and human Q fever incidence. However, there has been limited research on the broader range of risk factors influencing human Q fever incidence or on how urbanization affects these factors.

This study aimed to identify the major risk factors influencing human Q fever incidence in Korea and to examine how these factors differ between metropolitan cities and provinces. By applying both frequentist and Bayesian panel data analyses, it provides a comprehensive assessment of the determinants of Q fever incidence and elucidates epidemiological differences between regions. These findings are intended to support the development of tailored prevention and control strategies within the One Health framework.

## MATERIALS AND METHODS

### Data for evaluation of the human Q fever risk factors

This study examined Q fever incidence in Korea’s 17 metropolitan cities and provinces, using the annual incidence rate per 100,000 people as the dependent variable. Independent variables include the number of Q fever cases in livestock; the number of cattle, deer, and goats raised; and the number of livestock-related facilities (slaughterhouses, milk collection centers, feed factories, livestock markets, and artificial insemination [AI] centers) recorded at the end of each year. To ensure normality, livestock population counts were log-transformed. The dependent variable was obtained from the KDCA’s Infectious Disease Portal, while independent variables were sourced from the Korea Animal Health Integrated System (KAHIS) of the Ministry of Agriculture, Food and Rural Affairs (MAFRA). The dataset spans an 8-year period (2017-2024) and was structured as a panel dataset. Table 1 presents the annual Q fever incidence rates per 100,000 people by metropolitan city and province, and Table 2 summarizes the descriptive statistics of the independent variables.

An assessment of multicollinearity showed that the variance inflation factors for log-transformed cattle, deer, and goat populations were 25.58, 17.35, and 13.53, respectively. These values were well above the threshold of 10, indicating a multicollinearity issue. To address this, principal component analysis (PCA) was performed, integrating the 3 livestock variables into a single component reflecting their common variation patterns. The first principal component had a standard deviation (SD) of 1.68, an explained variance of 94.43%, and a cumulative variance of 94.43%, indicating strong explanatory power. The component loadings were 0.588 for cattle, 0.569 for deer, and 0.575 for goats.

To identify risk factors for human Q fever incidence, both frequentist and Bayesian panel regression analyses were employed. The frequentist approach provides an intuitive measure of the average effect of independent variables on the dependent variable and is particularly useful for analyzing within-panel variability; however, it has limitations in quantifying uncertainty and fully capturing differences between panels. In contrast, the Bayesian approach incorporates uncertainty probabilistically and models inter-panel variation hierarchically, enabling more nuanced inference. All analyses were conducted in R version 4.4.2 (R Foundation for Statistical Computing, Vienna, Austria) using the *car, plm, glmnet, lmtest*, and *brms* packages [16-20].

### Frequentist regression models for analysis

Panel data were analyzed to assess the determinants of human Q fever incidence using 3 frequentist models: pooled ordinary least squares (OLS), fixed effects (FE), and random effects (RE). The conceptual framework and equations for each model are described below.

#### OLS

The pooled OLS model treats all panel observations as part of a single regression, assuming that all data points share identical characteristics and ignoring any regional differences. In this approach, unique, time-invariant differences between provinces are not considered. Coefficients are estimated using the standard OLS method according to the following equation (1):


(1) $$y_{it}=\beta_{0}+\beta_{1}{\text{X}}_{it}+\varepsilon_{it}$$

where *y_it_* represents the dependent variable for entity *i* at time *t*, Χ_*it*_ denotes the independent variable, *β*_0_ and *β*_1_ are the regression coefficients, and *ε_it_* is the error term (*ε_it_*~*N*(0, *σ*^2^)). Although the pooled OLS model is straightforward to interpret and computationally efficient, it does not capture entity-specific differences, leading to potential endogeneity issues. Consequently, it does not fully leverage the advantages of panel data analysis.

#### FE model

The FE model accounts for entity-specific differences by removing time-invariant factors unique to each entity while focusing on analyzing the effects of independent variables that change over time [21]. To achieve this, the model includes an entity-specific effect (*α_i_*) in the estimation. The regression equation for the FE model is as follows:


(2) $$y_{it}=\beta_{0}+\beta_{1}{\text{X}}_{it}+\alpha_{i}+\varepsilon_{it}$$

where *α_i_* represents the unique characteristics of each metropolitan city and province, which are considered FE that do not change over time. The remaining symbols are the same as in the pooled OLS model. Using the FE model makes it possible to control for the influence of time-invariant factors, such as the average climate conditions and geographical characteristics of each municipality. That is, the model eliminates the impact of these factors on the dependent variable, enabling the estimation of the pure effect of time-varying independent variables on the dependent variable. However, since the FE model treats entity-specific characteristics as fixed values and removes them from the analysis, it cannot directly include variables that do not change over time. These variables are absorbed into the entity-specific effect (*α_i_*), meaning that their individual regression coefficients cannot be estimated separately, which is a limitation of this approach.

#### RE model

The RE model accounts for entity-specific differences, but considers them as probabilistic (random) variations. That is, while each entity has its own unique effect (*α_i_*), these effects are assumed to be random variables that follow a common distribution [21]. The regression equation for the RE model is as follows:


(3) $$y_{it}=\beta_{0}+\beta_{1}X_{it}+\left(\alpha_{i}+\varepsilon_{it}\right)$$

where *α_i_* ~*N*(0, *σ*^2^_α_), meaning that the individual RE follow a normal distribution. The RE model permits the inclusion of time-invariant variables. Additionally, since it has a higher degree of freedom than the FE model, its estimates may be more efficient. However, the model assumes that the entity-specific effects (*α_i_*) are independently distributed, and if this assumption is violated, the estimation results may be biased.

In this study, pooled OLS, FE, and RE models were applied sequentially. The Hausman test was used to determine whether the FE or RE model was more appropriate [22]. Additionally, the Breusch-Pagan Lagrange Multiplier (LM) test was performed to determine whether the pooled OLS or RE model was more suitable [23], and the Wooldridge test was conducted to check for autocorrelation in the FE model [24].

### Bayesian models for analysis

#### Bayesian fixed model for analysis

Unlike the frequentist approach described above, the Bayesian approach incorporates prior information (prior distributions) and quantifies uncertainty, offering distinct advantages. In particular, while frequentist estimates can become unstable when data are limited, the Bayesian method enables more stable inference in small-sample contexts by leveraging prior distributions [25]. The Bayesian FE model is a regression framework based on Bayesian inference that accounts for the unique characteristics of each municipality through FE [26]. In this model, prior distributions are assigned to each coefficient in equation (2), converting it into a fully probabilistic formulation.

#### Prior distribution

In the Bayesian FE model, prior distributions are assigned to *α_i_* and *β*_1_, while the error term (*ε_it_*) is assumed to follow a normal distribution:


$$\alpha i∼N\left(\mu_{\alpha },\sigma_{\alpha }^{2}\right),\;\beta_{1}∼N\left(\mu_{\beta },\sigma_{\beta }^{2}\right),\;\beta_{0}∼N\left(\mu_{\beta 0},\sigma_{\beta 0}^{2}\right),\;\varepsilon_{it}∼N\left(0,\sigma^{2}\right)$$

#### Likelihood

The likelihood function represents the probability of observing the given data for specified parameters. For the Bayesian FE model, it is expressed as:


(4) $$p\left(y{\left|\beta_{0},\beta_{1},\alpha ,\text{X},\sigma^{2}\right|}_{\text{y}}\right)=\prod_{i=1}^{N}\prod_{t=1}^{T}N\left(y_{it}|\beta_{0}+\beta_{1}{\text{X}}_{it}+\alpha_{i},\sigma^{2}\right)$$

#### Posterior distribution

In Bayesian methodology, Bayes’ theorem is used to compute the posterior distribution. The posterior distribution is obtained by combining the prior distribution with the likelihood function and is estimated through sampling using the Markov Chain Monte Carlo (MCMC) method [27]:


(5) $$p\left(\beta_{0},\beta_{1},\alpha ,\sigma^{2}|y,X\right)\propto p\left(y|\beta_{0},\beta_{1},\alpha ,X,\sigma^{2}\right)p\left(\beta_{0}\right)p\left(\beta_{1}\right)p(\alpha )p\left(\sigma^{2}\right)$$

In the frequentist FE model, estimating individual *α_i_* for many entities can reduce degrees of freedom, but in the Bayesian FE model, prior distributions for *α_i_* help prevent model overfitting [28]. This study’s Bayesian FE model does not consider RE across individual regions; instead, it estimates a single regression coefficient for all regions. A relatively weak normal prior was used, where the prior distribution for regression coefficients follows a normal distribution with a mean of 0 and a SD of 5, thereby avoiding unnecessary constraints in the absence of strong prior knowledge and encouraging data-driven inference [29]. For intercepts, which generally exhibit greater variability, a SD of 10 was used to allow a wider range. The likelihood was assumed to follow a Gaussian distribution.

MCMC sampling was conducted using 4 chains, each generating 5,000 samples (iteration=5,000). The first 1,500 samples were discarded as warm-up iterations (warmup=1,500), and all remaining samples were retained without thinning (thin=1), resulting in a total of 14,000 post-warmup draws used for analysis.

#### Bayesian hierarchical model for analysis

The Bayesian hierarchical model is a Bayesian inference-based model that reflects the multilevel structure of the data [30]. In this study, the 17 local governments were categorized into 2 groups: metropolitan cities and provinces. Since urbanized metropolitan cities and less urbanized provinces are likely to be influenced by different environmental and epidemiological factors, this hierarchical approach accounts for both within-group variation and between-group differences. In this framework, cities and provinces are modeled with group-level RE (random intercepts), allowing the model to capture structural differences between the 2 groups while still recognizing shared characteristics. This combination of FE and RE is particularly advantageous for datasets with nested subgroups that share common structures.

The Bayesian hierarchical model adopted the same prior distributions as the Bayesian FE model. However, for regional RE (random intercepts), a half-Cauchy(0,2) distribution was used to prevent excessive variability in the RE. The likelihood function and MCMC sampling conditions were set identically to those in the Bayesian FE model.

### Ethics statement

The incidence of Q fever was calculated using the infectious disease portal of the KDCA, and the number of livestock and livestock facilities were using the KAHIS data of the MAFRA. Due to the use of de-identified, data, this study was exempted from ethical review and informed consent.

## RESULTS

### Results of frequentist models

In this study, the dependent variable was the Q fever incidence per 100,000 population, while the independent variables included the number of Q fever cases in livestock; the number of susceptible livestock (cattle, deer, goats); and the number of livestock-related facilities (slaughterhouses, milk collection centers, feed factories, livestock markets, and AI centers). Considering the characteristics of panel data, pooled OLS, RE, and FE models were applied and compared (Table 3).

In the pooled OLS analysis, the coefficient for slaughterhouses was 1.20, indicating a significant positive association with Q fever incidence (p<0.001). Milk collection centers and feed factories showed negative associations. The model’s R² was 0.41, and the F-statistic p-value was<0.01, confirming overall model significance. In the RE model, slaughterhouses also demonstrated a significant positive effect (coefficient=1.03, p<0.01), while feed factories and AI centers showed negative effects. The R² for the RE model was 0.23. In the FE model, the coefficient for slaughterhouses was 0.14, indicating a positive but non-significant relationship. Livestock markets had a significant negative coefficient (-2.01, p<0.01). Although the R² for the FE model was relatively low at 0.12, the F-statistic p-value of 0.04 confirmed overall significance. To identify the most appropriate model, the Breusch-Pagan LM test and the Hausman test were performed. The LM test, comparing pooled OLS and RE models, yielded a chi-square statistic of 2.88 (p=0.09). Using a strict 5% significance threshold, pooled OLS was preferred; however, under a relaxed 10% threshold, the RE model could also be considered. The Hausman test produced a chi-square statistic of 16.62 (p=0.02), rejecting the null hypothesis (H₀) that the RE model was appropriate, and confirming the FE model as more suitable.

Despite the FE model’s appropriateness, a Wooldridge test revealed serial correlation (F-statistic=14.99, p<0.01), rejecting the H₀ of no serial correlation. This result indicated that the error terms of the FE model were not independent and were temporally correlated. To address this issue, the FE model was adjusted using Driscoll-Kraay robust standard errors (Table 3) [31]. After this adjustment, significant associations were observed for the number of Q fever cases in livestock (-0.11, p<0.01), livestock population (0.55, p<0.01), and livestock markets (-2.01, p<0.05) with human Q fever incidence.

### Results of Bayesian models

Before applying Driscoll-Kraay standard errors in the frequentist FE model, some variables, such as slaughterhouses, were significant in the pooled OLS and RE models but not in the FE model. This suggests that controlling for individual regional characteristics within panels can alter the estimated effects of certain variables. However, the frequentist framework provides only point estimates, making it difficult to capture uncertainty fully. Moreover, the FE model’s application to a small dataset reduced the degrees of freedom, increased standard errors, and lowered statistical significance. To overcome these limitations, a Bayesian FE model was implemented (Table 4).

To overcome these limitations, a Bayesian FE model was implemented (Table 4). The Bayesian framework quantifies uncertainty via posterior distributions, supports robust inference with small samples through prior information, and addresses issues such as autocorrelation and heteroscedasticity via MCMC sampling. The Bayesian analysis estimated the mean coefficient for slaughterhouses as 0.87 (95% credible interval [CrI], 0.21 to 1.42), indicating a clear positive effect. Other variables were not statistically significant, as their CrI included zero. By incorporating uncertainty directly, the Bayesian approach complemented the frequentist results and reinforced the interpretation of slaughterhouses as a determinant of Q fever incidence.

Since this study used panel data from 17 metropolitan cities and provinces, an additional analysis was conducted to capture differences between these 2 groups. Metropolitan cities and provinces likely differ in environmental factors such as livestock farming practices, industrial structure, and urbanization rate. To account for these potential differences, a Bayesian hierarchical model was applied (Table 5), incorporating cities/provinces as group-level RE.

The 17 local governments were classified into 2 groups: metropolitan cities and provinces. As in the Bayesian FE model, MCMC sampling was conducted. The results showed that slaughterhouses (95% CrI, 0.99 to 1.68) were a significant factor associated with increased human Q fever incidence, while feed factories (95% CrI, -0.54 to -0.19) and AI centers (95% CrI, -0.49 to -0.01) demonstrated negative associations. Although a model including a RE to distinguish between cities and provinces was applied, the potential for substantial differences between these groups prompted an additional separate analysis (Table 4). The findings indicated that the effect of slaughterhouses was stronger in metropolitan cities (1.46) than in provinces (1.22), suggesting a closer link between slaughterhouse activity and elevated Q fever incidence in metropolitan cities. In provinces, livestock population exerted a significant positive effect (0.94, 95% CrI, 0.15 to 1.74), indicating that greater livestock density may contribute to higher human Q fever incidence. Moreover, while milk collection centers, feed factories, and AI centers were not significant in metropolitan cities, they showed significant negative associations with Q fever incidence in provinces.

## DISCUSSION

This study, conducted from a One Health perspective, aimed to identify risk factors for human Q fever incidence by analyzing panel data from 17 metropolitan cities and provinces over an 8-year period (2017-2024). Key variables included the number of Q fever cases in livestock, the number of susceptible livestock, and the presence of livestock-related facilities such as slaughterhouses and feed factories. Both frequentist and Bayesian methodologies were applied. To address multicollinearity among the numbers of cattle, deer, and goats, PCA was used, allowing for a more integrated assessment of livestock density effects. Frequentist models compared FE and RE approaches, while Bayesian analysis was employed to quantify uncertainty probabilistically and to produce more reliable estimates of regional data distributions.

The results showed that slaughterhouses were not statistically significant in the FE model, but emerged as a key factor increasing Q fever incidence in the RE and Bayesian models. This discrepancy suggests that the FE model’s control of regional FE may have masked variability in slaughterhouse numbers, whereas the RE and Bayesian models were better able to capture their impact by incorporating inter-regional differences. Moreover, risk factors differed between highly urbanized metropolitan cities and less urbanized provinces. In metropolitan cities, slaughterhouses exerted a stronger effect, indicating the need for region-specific control measures. Preventive strategies for slaughterhouse workers and nearby residents could include mandatory mask use, targeted hygiene training, and routine Q fever antibody testing.

In contrast, the study found that in provinces, livestock density had a significant positive association with Q fever incidence, underscoring the need for proactive disease control in high-density livestock regions. Recommended measures include regular antibody testing of susceptible livestock, prompt isolation of infected animals, and improved dust and waste management in livestock facilities. Biosecurity training for farmers, livestock workers, and veterinarians—emphasizing the consistent use of protective clothing and adherence to personal hygiene—should also be strengthened. Previous studies indicate that over two-thirds of global Q fever cases occur outside traditional high-risk settings, with many cases in individuals who had no direct livestock contact. Indirect exposure, often via environmental contamination, has been identified as a frequent transmission pathway [5]. This suggests that *C. burnetii* can persist in the air for extended periods and disperse widely [32].

Due to the difficulty of acquiring highly detailed data at the municipal level, this study analyzed data at the metropolitan and provincial scale. This binary classification is justified by the substantial differences in demographic composition, land use, and livestock industry structure between the 2 levels, making it appropriate for both epidemiological and policy analysis. However, aggregating data at the administrative district level can obscure micro-level risk factors, as industrial complexes and livestock farms may coexist within the same administrative boundaries. Such spatial aggregation can introduce bias, potentially underestimating the influence of specific risk factors or failing to capture localized patterns of Q fever incidence. Future research should employ more granular spatial units—such as municipalities, townships, or individual facilities—to better capture intra-regional heterogeneity and localized risk determinants.

The observed decline in Q fever incidence since 2020 may, in part, reflect the indirect impact of the coronavirus disease 2019 (COVID-19) pandemic [4]. This means the downward trend does not necessarily indicate a reduction in the underlying risk of Q fever infection, and recent patterns should be interpreted cautiously. These findings carry important policy implications. Given the high proportion of asymptomatic Q fever cases, early detection is challenging [33]. Increasing awareness among clinicians and local communities, along with improving diagnostic accessibility, is essential. Furthermore, enhanced surveillance of Q fever in animals and improved information sharing between human and veterinary health sectors are critical components of a One Health-based response.

As surveillance data for both human and animal cases continue to accumulate, and as more granular statistics on Q fever risk factors become available at finer spatial resolutions, the continued application of Bayesian methods is expected to further reduce uncertainty and facilitate the development of more targeted and effective prevention and management strategies.

## Figures and Tables

**Figure f1-epih-47-e2025046:**
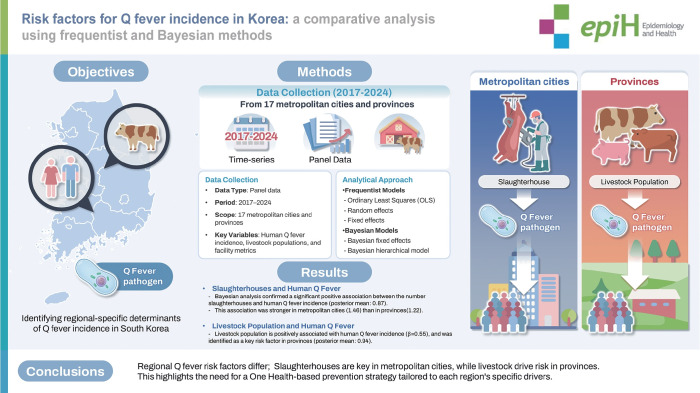


**Table 1.** Q fever incidence per 100,000 people in Korea (2017-2024)^1^

Areas		2017	2018	2019	2020	2021	2022	2023	2024
Total		0.19	0.31	0.31	0.13	0.09	0.11	0.11	0.11
Metropolitan cities	Seoul	0.05	0.15	0.06	0.01	0.06	0.00	0.05	0.05
	Busan	0.03	0.09	0.00	0.03	0.09	0.00	0.06	0.03
	Daegu	0.04	0.08	0.33	0.00	0.00	0.13	0.13	0.13
	Incheon	0.07	0.10	0.10	0.10	0.03	0.03	0.10	0.07
	Gwangju	0.14	0.68	0.55	0.14	0.07	0.14	0.00	0.07
	Daejeon	0.27	0.20	0.54	0.20	0.48	0.35	0.35	0.07
	Ulsan	0.09	0.69	0.09	0.00	0.18	0.00	0.00	0.09
	Sejong	0.00	0.00	0.00	0.29	0.00	0.26	0.00	0.00
Provinces	Gyeonggi	0.07	0.18	0.14	0.08	0.01	0.05	0.10	0.07
	Gangwon	0.00	0.06	0.00	0.00	0.00	0.07	0.00	0.00
	Chungbuk	1.95	1.75	1.88	0.75	0.31	0.56	0.63	0.44
	Chungnam	0.62	0.94	0.94	0.47	0.38	0.42	0.24	0.19
	Jeonbuk	0.22	0.38	0.71	0.33	0.17	0.28	0.06	0.11
	Jeonnam	0.37	1.11	1.23	0.70	0.11	0.22	0.17	0.17
	Gyeongbuk	0.15	0.26	0.52	0.04	0.19	0.19	0.16	0.55
	Gyeongnam	0.36	0.33	0.27	0.15	0.03	0.12	0.06	0.09
	Jeju	0.00	0.15	0.00	0.00	0.00	0.00	0.00	0.00

1 Q fever incidence data were obtained from the Infectious Disease Portal of the Korea Centers for Disease Control and Prevention.

**Table 2.** Descriptive statistics of independent variables^1^ by metropolitan city and province, Korea (2017-2024)

Areas	Livestock Q fever cases	No. of cattle	No. of deer	No. of goats	No. of slaughterhouses	No. of dairy platforms	No. of feed factories	No. of livestock market	No. of AI facilities
Seoul	0.0±0.0	2,342±78	29±18	24±23	0.0±0.0	0.0±0.0	0.0±0.0	0.0±0.0	0.0±0.0
Busan	0.1±0.4	2,583±66	19±11	97±35	1.0±0.0	0.0±0.0	0.0±0.0	0.0±0.0	0.0±0.0
Daegu	0.1±0.3	1,388±71	26±8	1,315±57	0.0±0.0	0.0±0.0	0.0±0.0	0.0±0.0	0.0±0.0
Incheon	0.1±0.4	4,561±75	94±17	279±30	1.1±0.4	0.1±0.4	0.1±0.3	0.0±0.0	0.0±0.0
Gwangju	0.4±0.8	2,081±88	22±10	5±7	1.0±0.0	0.0±0.0	0.0±0.0	0.0±0.0	0.0±0.0
Daejeon	0.3±0.7	1,481±110	48±23	134±16	1.0±0.0	0.0±0.0	0.0±0.0	0.0±0.0	0.0±0.0
Ulsan	0.0±0.0	2,815±44	13±8	68±19	1.0±0.0	0.0±0.0	0.0±0.0	0.0±0.0	0.0±0.0
Sejong	0.0±0.0	1,405±71	9±7	165±18	0.0±0.0	0.0±0.0	0.0±0.0	0.0±0.0	0.0±0.0
Gyeonggi	0.8±0.7	43,484±968	395±112	1,122±308	4.8±0.4	2.9±0.3	2.8±0.3	1.4±0.2	1.3±0.2
Gangwon	0.0±0.0	41,568±703	1,480±337	721±71	3.0±0.0	2.5±0.4	1.4±0.3	0.4±0.2	0.7±0.1
Chungbuk	11.1±16.6	69,743±3,336	4,012±1,701	7,448±2,064	7.5±0.7	7.1±0.6	3.8±0.2	1.3±0.2	2.8±0.2
Chungnam	6.3±6.6	120,339±1,947	6,217±1,174	13,063±2,088	13.5±0.5	9.3±0.8	8.5±0.6	2.3±0.2	4.9±0.3
Jeonbuk	2.1±2.0	66,124±1,712	1,723±140	9,473±1,442	10.6±0.5	6.1±0.5	6.0±0.2	1.9±0.2	3.0±0.2
Jeonnam	3.6±4.5	85,090±2,186	8,543±1,547	14,020±2,523	14.5±0.8	7.3±0.8	7.9±0.5	2.0±0.2	4.5±0.4
Gyeongbuk	3.8±6.3	98,743±2,401	10,654±2,209	15,477±2,543	17.0±0.7	6.6±0.7	6.4±0.4	2.5±0.2	4.5±0.4
Gyeongnam	2.6±2.3	70,412±1,656	5,058±800	8,492±1,374	9.4±0.5	5.3±0.4	6.0±0.2	1.8±0.1	3.5±0.2
Jeju	0.0±0.0	7,747±70	113±15	25±9	2.0±0.0	0.0±0.0	0.0±0.0	0.0±0.0	0.0±0.0

Values are presented as mean±standard deviation. AI, artificial insemination.

1 Independent variable data were obtained from the Ministry of Agriculture, Food and Rural Affairs’ Korea Animal Health Integrated System.

**Table 3.** Panel regression analysis results of pooled OLS, FE, and RE models^1^

Variables	Pooled OLS		RE		FE		FE Driscoll-Kraay	
	Estimate	SE	Estimate	SE	Estimate	SE	Estimate	SE
Intercept	0.00	0.07	0.00	0.10	-	-	-	-
No. of livestock Q fever cases	0.02	0.07	-0.03	0.07	-0.11	0.07	-0.11^**^	0.04
No. of livestock (PCA)	-0.04	0.08	0.05	0.11	0.55	0.42	0.55^**^	0.18
No. of slaughterhouses	1.20^***^	0.16	1.03^**^	0.22	0.14	0.41	0.14	0.24
No. of dairy platforms	-0.25^*^	0.12	-0.27	0.17	-0.44	0.50	-0.44	0.25
No. of feed factories	-0.35^***^	0.09	-0.27^*^	0.11	0.08	0.18	0.08	0.11
No. of livestock markets	-0.22	0.15	-0.20	0.20	-2.01^**^	0.75	-2.01^*^	1.01
No. of AI facilities	-0.24	0.12	-0.26^*^	0.13	-0.02	0.17	-0.02	0.18
R^2^	0.41		0.23		0.12			
F-statistic	12.58		-		2.17			
*χ* ^2^	-		39.27		-			
p-value	<0.01		<0.01		0.04			

OLS, ordinary least squares; FE, fixed effects; RE, random effects; SE, standard error; PCA, principal component analysis; AI, artificial insemination.

1 Model selection tests were conducted to determine the most appropriate specification; The Breusch-Pagan Lagrange Multiplier test (χ²=2.88, p=0.09) indicated that both the pooled OLS and RE models were plausible under a relaxed 10% threshold; However, the Hausman test (χ²=16.62, p=0.02) rejected the null hypothesis of RE suitability, confirming that the FE model was more appropriate; The Wooldridge test (F=14.99, p<0.01) suggested the presence of autocorrelation in the FE model, and thus, Driscoll-Kraay robust standard errors were applied.

* p<0.05,

** p<0.01,

*** p<0.001.

**Table 4.** Bayesian model analysis results^1^

Variables	Bayesian fixed model				Bayesian hierarchical model			
	Estimate	SE	95% CrI		Estimate	SE	95% CrI	
			LL	UL			LL	UL
Intercept	0.00	0.14	-0.28	0.29	-0.04	1.47	-3.21	2.82
No. of livestock Q fever cases	-0.04	0.07	-0.18	0.11	0.00	0.07	-0.14	0.15
No. of livestock (PCA)	0.15	0.18	-0.16	0.56	0.01	0.08	-0.15	0.18
No. of slaughterhouses	0.87	0.31	0.21	1.42	1.34	0.17	0.99	1.68
No. of dairy platforms	-0.29	0.23	-0.76	0.15	-0.21	0.13	-0.46	0.03
No. of feed factories	-0.21	0.14	-0.47	0.09	-0.37	0.09	-0.54	-0.19
No. of livestock markets	-0.25	0.26	-0.81	0.24	-0.12	0.16	-0.42	0.19
No. of AI facilities	-0.25	0.14	-0.52	0.02	-0.25	0.12	-0.49	-0.01
SD (intercept)	0.49	0.19	0.18	0.93	1.52	1.73	0.15	6.24
Sigma	0.73	0.05	0.64	0.84	0.78	0.05	0.69	0.89

SE, standard error; CrI, credible interval; LL, lower limit; UL, upper limit; PCA, principal component analysis; AI, artificial insemination; SD, standard deviation.

1 Draws: 4 chains, each with iteration=5,000; Burn in=1,500; Thin=1; Total post-warmup draws=14,000.

**Table 5.** Bayesian hierarchical model analysis results for metropolitan cities versus provinces^1^

Variables	Metropolitan cities				Provinces			
	Estimate	SE	95% CrI		Estimate	SE	95% CrI	
			LL	UL			LL	UL
Intercept	-2.06	1.36	-4.74	0.64	-1.04	0.33	-1.70	-0.38
No. of livestock Q fever cases	-0.05	0.21	-0.45	0.36	-0.02	0.09	-0.21	0.16
No. of livestock (PCA)	-0.01	0.06	-0.13	0.12	0.94	0.41	0.15	1.74
No. of slaughterhouses	1.46	0.56	0.34	2.57	1.22	0.23	0.77	1.67
No. of dairy platforms	-0.43	0.46	-1.34	0.46	-0.46	0.2	-0.86	-0.06
No. of feed factories	-1.88	1.76	-5.31	1.56	-0.33	0.11	-0.54	-0.11
No. of livestock markets	-3.06	1.81	-6.60	0.51	-0.46	0.25	-0.96	0.04
No. of AI facilities	0.55	1.35	-2.12	3.21	-0.29	0.15	-0.58	-0.01
Sigma	0.46	0.04	0.38	0.56	0.96	0.09	0.81	1.15

SE, standard error; CrI, credible interval; LL, lower limit; UL, upper limit; PCA, principal component analysis; AI, artificial insemination.

1 Draws: 4 chains, each with iter=5,000; Warmup=1,500; Thin-1; Total post-warmup draws=14,000.
